# P-206. Change in Cryptococcal Antigen Titer Over Time in Uganda

**DOI:** 10.1093/ofid/ofaf695.428

**Published:** 2026-01-11

**Authors:** Alexandra Poeschla, Radha Rajasingham, Abduljewad Wele, Elizabetha Nalintya, Biyue Dai, David Meya, David R Boulware

**Affiliations:** University of Minnesota, St. Paul, MN; University of Minnesota, St. Paul, MN; University of Minnesota, St. Paul, MN; Infectious Disease Institute, Kampala, Kampala, Uganda; University of Minnesota, St. Paul, MN; Infectious Diseases Institute, Makerere University, Kampala, Kampala, Uganda; University of Minnesota, St. Paul, MN

## Abstract

**Background:**

Cryptococcal meningitis causes an estimated 19% of AIDS-related deaths globally and is the leading cause of meningitis in adults with HIV. Subclinical infection with cryptococcal antigen (CrAg) is detectable in plasma, and CrAg titer of >= 1:160 is associated with increased risk of meningitis or death. We evaluated if plasma CrAg titer changed over time in Uganda due to the expansion of national cryptococcal screening programs and increased access to antiretroviral therapy (ART).

**Figure:**
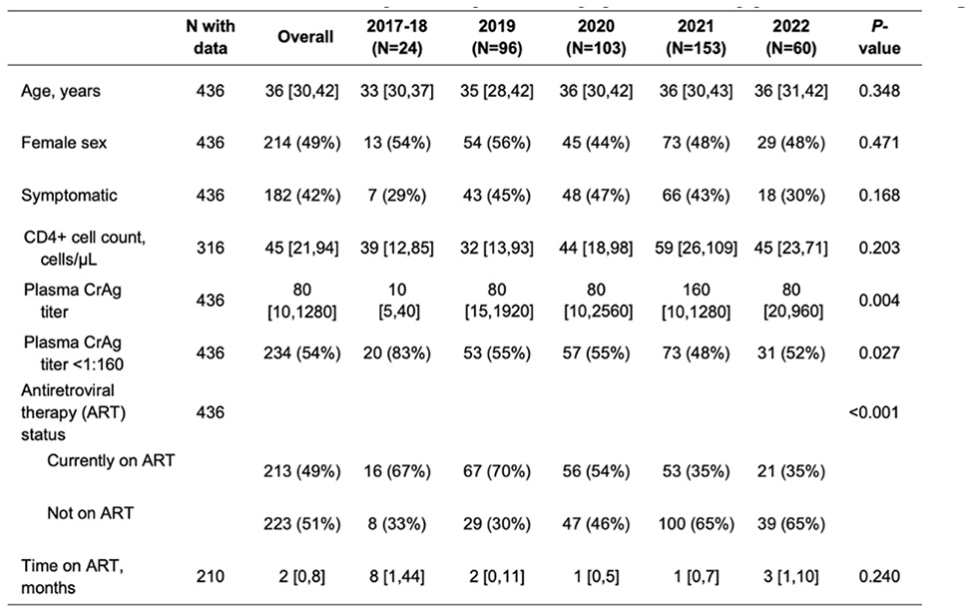
Baseline characteristics of participants by year of cryptococcal antigen screening. Data are presented as n (%) or median [IQR]. Fisher exact test was used to compare proportions and Kruskal-Wallis test was used for medians. Abbreviations: ART, antiretroviral therapy; CrAg, cryptococcal antigen; IQR, interquartile range.

**Figure:**
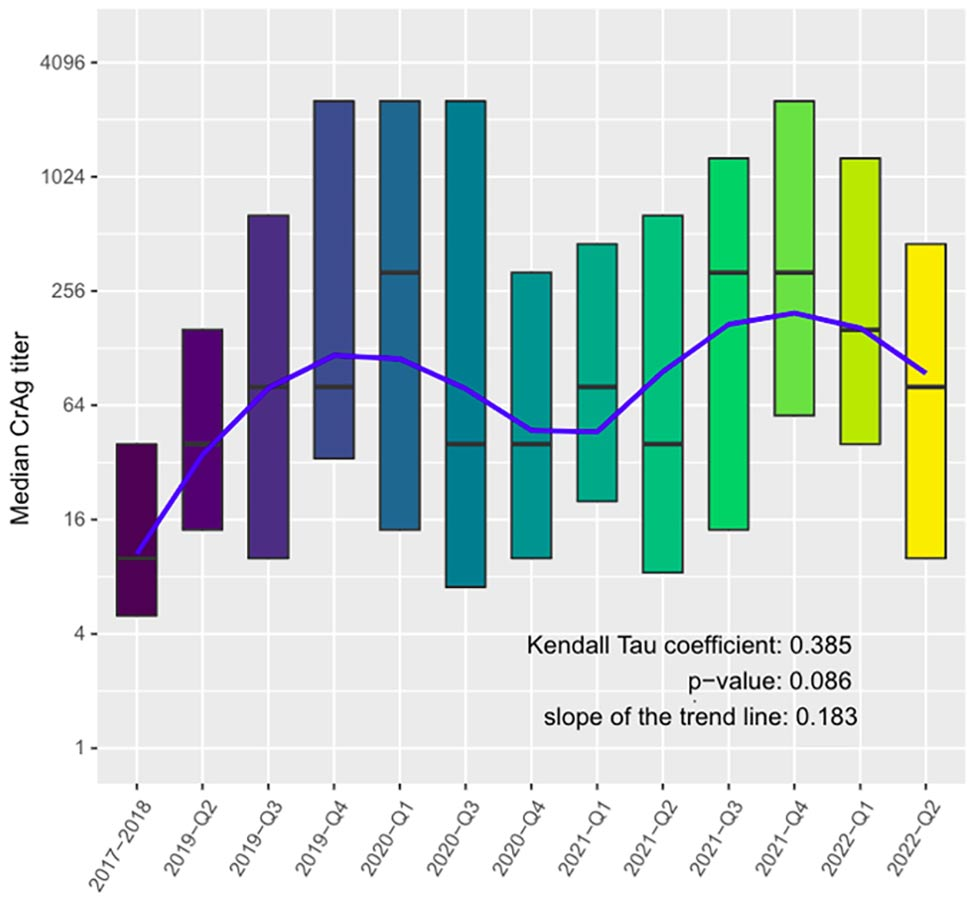
Trend in baseline median CrAg titer from 2017-2022 by quartiles. The blue line represents the estimated Sen's trend.

**Methods:**

We prospectively screened adults with advanced HIV disease for plasma CrAg using the lateral flow assay and performed titers. We assessed median plasma CrAg titer and interquartile range by year and quartile from 2017 through 2022. We used Fisher’s exact test to compare the proportion with a high CrAg titer ( >= 1:160) over time in addition to other demographic characteristics. We applied the Mann-Kendall trend test with Sen’s estimate to analyze trends in median titers.

**Results:**

From November 2017 to May 2022, 436 adults with advanced HIV disease had a positive plasma CrAg test. The median CD4+ cell count was 45 [IQR: 21,94] cells/µL, and median plasma CrAg titer was 1:80 [IQR: 10,1280]. Analysis of median quarterly CrAg titer from 2017-2022 demonstrated a non-statistically significant positive trend in CrAg titer (tau=0.385, p=0.086). The proportion of those on ART significantly decreased over time. No significant changes occurred in median CD4 count, proportion by sex, or duration of ART.

**Conclusion:**

Despite expansion of CrAg screening programs and ART access in Uganda, median annual CrAg titer has not decreased between 2017 and 2022. Cryptococcosis persists in Uganda, and despite public health efforts, people are not presenting to care earlier in their disease course. In addition, the proportion on ART decreased over time. Continued expansion and refinement of CrAg screening programs and access to ART among individuals with HIV is needed in Uganda, to reduce and ultimately eliminate AIDS-related deaths. Further analysis of CrAg titer trends in other countries with a high burden of HIV would provide more insight into whether national screening programs result in earlier identification of CrAg-positive individuals, and lower plasma CrAg titer.

**Disclosures:**

All Authors: No reported disclosures

